# Development of a Highly Sensitive SPR Biosensor for BCR–ABL Gene Sequence Detection Using a Novel Gold Nanoparticle–Enhanced Sandwich Assay Format

**DOI:** 10.3390/mi17040426

**Published:** 2026-03-30

**Authors:** Maksym S. Sobolevskyi, Andrii M. Lopatynskyi, Anton V. Samoylov, Glib V. Dorozinsky, Oleksandr M. Lyapin, Roman V. Khrystosenko, Volodymyr I. Chegel, Viktoriya M. Pyeshkova, Abdelhamid Errachid, Sergei V. Dzyadevych, Oleksandr O. Soldatkin

**Affiliations:** 1 Institute of Molecular Biology and Genetics, National Academy of Sciences of Ukraine, 150 Zabolotnoho Str., 03680 Kyiv, Ukraine; maxim.sobolevskiy@gmail.com (M.S.S.); dzyad@yahoo.com (S.V.D.); soldatkinsasha@gmail.com (O.O.S.); 2 Lashkaryov Institute of Semiconductor Physics, National Academy of Sciences of Ukraine, 41, Nauki Avenue, 03028 Kyiv, Ukraine; lop2000@ukr.net (A.M.L.);; 3 Educational Scientific Institute of High Technologies, Taras Shevchenko National University of Kyiv, 64 Volodymyrska Str., 01003 Kyiv, Ukraine; 4 School of Engineering, University of Warwick, Coventry CV4 7AL, UK; 5 Universite Claude Bernard Lyon 1, Institut des Sciences Analytiques, Unité Mixte de Recherche 5280, Centre National de la Recherche Scientifique, 5 rue de la Doua, 69100 Villeurbanne, France; 6 National Technical University of Ukraine “Igor Sikorsky Kyiv Polytechnic Institute”, Department of Translational Medical Bioengineering, 37 Beresteysky Avenue, 03056 Kyiv, Ukraine

**Keywords:** DNA hybridization-based biosensors, BCR–ABL fusion gene, DNA sandwich assay, surface plasmon resonance, gold nanoparticles, AuNP-labeled biosensor

## Abstract

SPR (surface plasmon resonance) biosensor–based analytical methods enable rapid, straightforward, and cost-effective detection of DNA oligonucleotides. However, the detection limits of currently available SPR biosensors for BCR–ABL gene oligonucleotides remain too high to reliably detect sub-nanomolar concentrations. This study presents a new signal-enhancement approach for SPR DNA biosensors based on a gold nanoparticle (AuNP) sandwich assay. In this work, we demonstrated that AuNP-modified oligonucleotides can serve as labels that significantly amplify the SPR biosensor response in a sandwich-type SPR DNA biosensor. The analytical characteristics of the developed AuNP-labeled biosensor for detection of BCR–ABL fusion gene oligonucleotides were studied. The AuNP-labeled biosensor exhibited a detection limit of 80 pM, which is significantly lower than that of a traditional label-free SPR biosensor (50 nM). The measurement error for BCR–ABL target detection was significantly lower with the AuNP-labeled biosensor than with the label-free SPR biosensor. The conditions of synthesis of AuNPs by citrate reduction of AuCl_3_ that allow the monodisperse size distribution and absence of AuNP aggregation were established as well. Based on the obtained data, we conclude that a sandwich assay employing AuNP-modified oligonucleotides as labels is a promising approach for the highly sensitive detection of genetic markers. The developed AuNP-labeled DNA biosensing approach can be adapted to enhance the signal in other DNA hybridization-based SPR biosensors.

## 1. Introduction

Surface plasmon resonance spectroscopy (SPR) biosensors based on DNA hybridization are important tools for the detection of intermolecular interactions in modern analytical and biomedical applications [1,2,3]. However, a key limitation for their broader medical application is their relatively high detection limit. Overcoming this challenge remains an active area of research and has stimulated advances in material science, chemistry, physics, and data analysis methods, including machine learning [4,5]. In particular, the analytical characteristics of SPR biosensors can be significantly improved through the use of gold nanoparticle (AuNP)–modified oligonucleotides as signal-enhancing probes [1,6,7].

Nanoparticles have been widely incorporated into many novel biosensors to improve their analytical performance [8,9,10,11,12,13,14,15]. To date, the application of gold nanoparticles has enabled substantial progress in the detection of biomolecules at physiologically relevant concentrations, including targets such as circulating tumor DNA (ctDNA) and microRNA (miRNA) [6,7,8]. AuNPs are particularly attractive for biosensing applications due to their high molecular weight, large surface area, ease of surface functionalization and unique optical properties, including intense light absorption and scattering, which are directly associated with localized surface plasmon resonances (LSPR). Owing to the short decay length of the localized plasmon evanescent field, this phenomenon is used in LSPR biosensors for the detection of biomolecules smaller than 10 nm. In contrast, the selective detection of larger molecules, such as oligonucleotides, can be achieved using SPR biosensors, in which the evanescent field penetration depth may reach up to 200 nm [13]. In such biosensors, biofunctionalized colloidal nanoparticles may be utilized to track biomolecular interactions at the sensor surface through coupling between the surface plasmons of the SPR sensor and the localized surface plasmons of the surface-bound nanoparticles. This enables the assay to take advantage of the strong local electric field associated with localized surface plasmons, thereby improving the detection of large ssDNA oligonucleotides [16].

This study is focused on the development of a sandwich assay for detection of the hybrid gene BCR–ABL, which currently is the object of extensive genetic and molecular biological studies due to it being a molecular marker of asymptomatic leukemia [17]. Quantitative PCR (qPCR) remains the gold standard for BCR–ABL detection due to its extremely high sensitivity and standardized reporting on the International Scale (IS), enabling detection of transcript levels as low as ~0.003% IS. In contrast, SPR-based biosensors provide real-time detection of nucleic acid hybridization, offering advantages in assay simplicity and the potential for rapid and inexpensive analysis. However, SPR detection limits are typically reported in absolute concentration units (e.g., nM–pM), which are not directly comparable to %IS values. Approximate order-of-magnitude considerations indicate that conventional SPR detection limits (e.g., ~10 nM) correspond to relatively high target abundances, significantly above the clinically relevant transcript levels measured by qPCR. Nevertheless, the incorporation of signal amplification strategies, such as nanoparticle-enhanced SPR, can substantially improve sensitivity toward the pM and sub-pM range, narrowing the gap with PCR-based methods and highlighting the potential of SPR biosensors for molecular diagnostics [18,19,20]. A comparison of qPCR assays and label-free SPR biosensor analysis for the detection of BCR–ABL sequences is presented in Table 1.

Based on the above, the purpose of this work was to develop a new technology for multiple amplification of the DNA hybridization-based SPR biosensor signal using AuNP-modified DNA molecules as labels in the sandwich analysis format for the detection of BCR–ABL gene oligonucleotides. In this approach, the sensor response is enhanced through the attachment of specially designed AuNP-conjugated DNA molecules to DNA duplexes formed between surface-immobilized probes and target oligonucleotides. This assay format, known as a sandwich assay, involves the formation of a ternary complex between the probe ligand, the target molecule, and the labeled ligand.

## 2. Materials and Methods

### 2.1. Reagents

The reagents used in this research were as follows: urea, KH_2_PO_4_, SSC buffer solution, lipoic acid (LA) and 6-mercapto-1-hexanol (MCH) (Fluka, Buchs, Switzerland), oligonucleotides (Metabion, Planegg, Germany), aurum (III) chloride, and dilute HCL (Thermo Fisher Scientific, Waltham, MA, USA); all reagents were of Pro Analysi quality. All solutions were prepared using Milli-Q deionized water.

### 2.2. Hybridization of Oligonucleotides in the Biosensor System

The sandwich assay is formed on the sensor surface in the measuring cell of the SPR spectrometer through affinity interactions involving three ssDNA molecules attributed to the e13a2 variant of BCR–ABL junction site, which is one of the most clinically relevant variants of the hybrid gene [24]. The ssDNA molecules are as follows: oligonucleotide-probe mod-Ph (SH-(CH_2_)_6_-GCT GAA GGG CTT TTG AAC TCT GCT), complementary to the 24-base region of the target oligonucleotide 80-mer BCR–ABL (TCA TCG TCC ACT CAG CCA CTG GAT TTA AGC AGA GTT CAA AAG CCC TTC AGC GGC CAG TAG CAT CTG ACT TTG AGC CTC AG), and the second oligonucleotide SH-DP (SH-(CH_2_)_6_-TTT TTT TTT GGC TGA GTG GAC GAT GA), complementary to another region of the 80-mer BCR–ABL target, with a length of 18 nitrogenous bases. The mod-Ph probe is immobilized on the sensor surface of the SPR spectrometer, and the SH-DP probe is immobilized on the surface of gold nanoparticles, facilitating their approach to the SPR sensor surface if 80-mer BCR–ABL is present on it.

Accordingly, if the 80-mer BCR–ABL sequence is introduced into the measuring flow cell, two hybridization interactions take place, after which the sensor response of the analyzer is measured by comparing the signal level of the biosensor before and after the introduction of the target solution with the 80-mer BCR–ABL DNA sequence in it.

### 2.3. Synthesis and Characterization of AuNPs

The production of AuNPs was carried out according to one of two different modifications of Turkevich’s method [25]. First, 20 mL of 1 mM HAuCl_4_ or AuCl_3_ was heated to boiling with constant stirring (solutions of HAuCl_4_ were prepared by the addition of 200 mM HCL to AuCl_3_ in a 1:1 molar ratio). Then, 2 mL of 1% (38.7 mM) sodium citrate was added. Heating and stirring were continued for several minutes until the colloid began to turn deep red, indicating the reduction of most of the gold ions [26].

The synthesized AuNPs were stored at a temperature of 4 °C. According to Dong et al., AuNPs obtained using HAuCl_4_ and sodium citrate in a molar ratio higher than 2.4 appear as practically monodisperse spherical structures with a size of about 10–20 nm, which are stabilized by citrate ions [27]. The AuNPs used in this work were characterized by a light absorption peak at approximately 520 nm [28,29,30,31].

Diameters of the studied AuNPs were evaluated with the photon correlation spectroscopy system of the Malvern Zetasizer Nano series. TEM images of the studied AuNPs were taken using a JEM-1400 transmission electron microscope (Jeol, Japan).

Concentrations of the studied AuNPs were measured through the Bouguert–Lambert–Beer law: A = εCL, where A is the optical density, ε is the molar extinction coefficient, C is the concentration, and L is the optical path length. To calculate the molar extinction coefficient of AuNPs, the method of Liu et al. was utilized [32,33].

### 2.4. Formation of Probe DNA-AuNP Complexes and Their Subsequent Colloidal Stabilization

The binding of thiolated oligonucleotides to gold is a well-established method for immobilizing the DNA probe on a gold sensor surface [23]. The high affinity of thiol groups for gold promotes the formation of stable covalent Au–S bonds. In the case of citrate-capped AuNPs, this enables controlled surface functionalization by replacing electrostatically bound citrate ions with covalently bound thiolated molecules [34].

The modification of thiolated oligonucleotides with AuNPs and the subsequent attachment of blocking molecules (MCH and LA) to such AuNPs was carried out by incubating 750 nM SH-DP oligonucleotides in a sol of 2.5 nM AuNPs for 12 h in 0.1 × SSC medium (1.5 mM trisodium citrate, 15 mM NaCl, pH 7.0) at room temperature, after which a solution of MCH and LA in 0.1 × SSC buffer solution was added to the formed conjugates until the concentration of both MCH and LA was equal to 2 μM. The resulting solution was incubated for 1 h at room temperature to achieve immobilization of MCH and LA on the free surface of AuNPs. Next, the AuNPs were transferred to the medium of Milli-Q deionized water by two 30-min centrifugations at a relative centrifugal force of 13,226 relative units, followed by discarding the supernatant. The molar concentration of thiolated oligonucleotides was selected based on the calculation of its ratio to the concentration of AuNPs at the level of 300:1 [35]. The use of lower probe-to-AuNP ratios is known to be connected to low colloidal stability in the resulting DNA-AuNP complexes in SSC media of high multiplicity [36]. The resulting colloid of SH-DP probe oligonucleotides modified with AuNPs stabilized with MCH and LA is further referred to as AuNP-SH-DP.

### 2.5. Design of a Biosensor System

**Figure 1:**
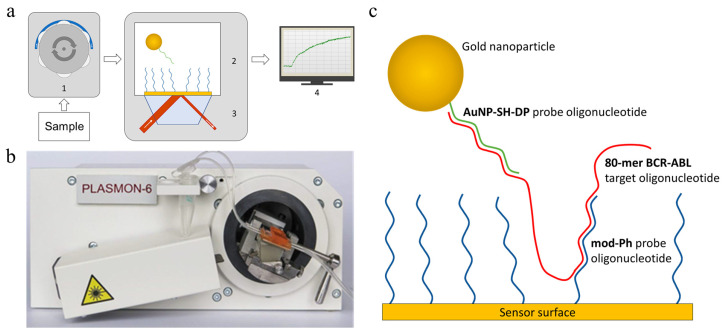
(**a**) Block diagram of the SPR biosensor system: peristaltic pump (1), bioselective element (2), SPR spectrometer (3), and computer with signal-processing software (4). (**b**) SPR spectrometer “Plasmon-6”. (**c**) Schematic of the sandwich assay used in the developed DNA hybridization–based SPR biosensor (AuNP-labeled SPR biosensor).

The investigation of oligonucleotide hybridization processes was carried out on a two-channel SPR spectrometer “Plasmon-6” in a Kretchmann optical configuration (Figure 1a,b), which was developed at the V.E. Lashkaryov Institute of Semiconductor Physics of the National Academy of Sciences of Ukraine [37]. The sensor surface of this device is a layer of gold with a thickness of 50 nm on a plate made of optical glass; the volume of the analytical cell was 120 μL. Before gold modification, the surface of the glass plate (chip) was cleaned with freshly prepared Piranha solution (3:1 mixture of concentrated H_2_SO_4_ and 30% H_2_O_2_; caution: Piranha solution is extremely corrosive, prone to decomposition and emits heat when prepared) at room temperature for 3 min, then thoroughly washed with distilled and deionized water and air-dried.

The cleaned chip was mounted on the spectrometer prism using an immersion fluid having the same refractive index as the prism and the chip. The flow rate (typically 40 μL/min) through the measuring flow cell was controlled by a peristaltic pump (Ismatec, USA) [38]. The SPR signal was processed using the Biosuplar software developed by Mivitec GmbH. All experiments were conducted at room temperature (+22 °C).

### 2.6. The Process of Oligonucleotide Immobilization on the Sensor Surface and Subsequent DNA Hybridization, with a Description of the Sandwich Assay

**Figure 2:**
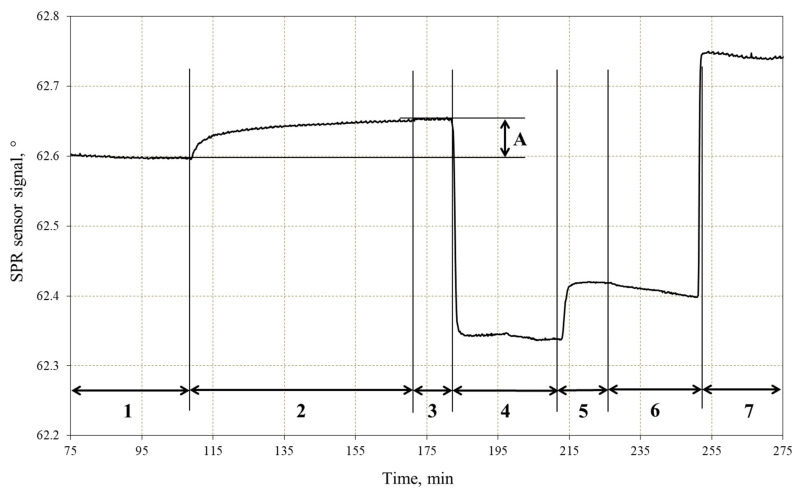
Typical immobilization sensorgram with the experimental stages indicated. *x*-axis: time (min); *y*-axis: resonance angle (°). 1—washing with the immobilization buffer (0.5 M KH_2_PO_4_, pH 3.8); 2—injection of 1 μM thiolated oligonucleotide probe (mod-Ph), followed by stop-flow incubation; 3—washing with the immobilization buffer; 4—washing with Milli-Q water; 5—injection of MCH, followed by stop-flow incubation; 6—washing with Milli-Q water; 7—washing with 2 × SSC buffer solution. A—immobilization level.

The preparation of a bioselective DNA sensor element includes two main stages: immobilization of the oligonucleotide probe and passivation of the free surface of the chip (Figure 2). To immobilize the thiolated probe, 1 μM mod-Ph in 0.5 M KH_2_PO_4_ (pH 3.8) was introduced into the measuring flow cell and incubated for 1 h. The mod-Ph probes were fully complementary to the 80-mer BCR–ABL target. After that, the areas on the gold surface that are free of immobilized mod-Ph were blocked with 0.1 mM aqueous solution of MCH [39].

At the beginning of each 80-mer BCR–ABL detection experiment, a stable baseline is first established by thoroughly washing the gold sensor surface, functionalized with mod-Ph probes, with 2 × SSC running buffer solution (30 mM sodium citrate, 300 mM NaCl, pH 7). All subsequent experiments are carried out in the same buffer solution. Next, the test sample containing the 80-mer BCR–ABL oligonucleotide in a certain concentration is introduced into the measuring flow cell and incubated for 10 min, which leads to the biosensor response. After that, to enhance the sensor response to the 80-mer BCR–ABL target, AuNP-modified SH-DP probes (AuNP–SH–DP) are introduced at a concentration of 450 pM, and the biosensor is incubated until the molecular sandwich complex forms via hybridization (Figure 1c). The greater the number of 80-mer BCR–ABL targets from the sample hybridizes with the mod-pH oligonucleotides, the more AuNP–SH–DP probes are captured in the resulting sandwich complex, leading to a higher SPR signal. The sensor response to the sample with the ssDNA target is determined by subtracting the SPR signal recorded before sample introduction from the SPR signal measured after sample addition, subsequent incubation with the AuNP–SH–DP probes, and completion of the sandwich complex formation.

To enable reuse of the DNA sensor, the hybridized DNA duplex is dissociated by treatment with an 8 M urea solution. Regeneration efficiency is assessed by comparing the SPR baseline recorded after the urea treatment with the initial baseline.

Standard methods of variational statistics were used to process the experimental data obtained during the work. Studies were performed in at least 3 to 5 replicates. In the statistical processing of the results, the arithmetic mean and its standard deviation were determined, and the data were considered significant at *p* < 0.05. Data processing and calculations were performed using the OriginLab software and Microsoft Excel [40]. Secondary structures of DNA molecules and Gibbs free energy ΔG values were calculated using the DINAMelt web server [41].

## 3. Results and Discussion

**Figure 3:**
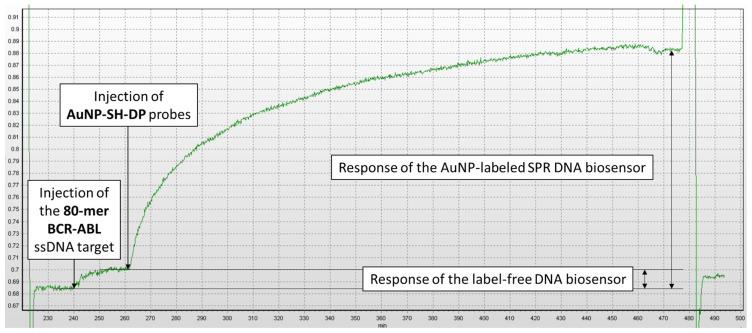
Real-time response of the SPR biosensor to the introduction of 800 nM solution of 80-mer BCR–ABL target. Hybridization medium—2 × SSC buffer solution, concentration of AuNP-SH-DP—450 pM. *y*-axis: SPR spectrometer signal (degrees, °); *x*-axis: time (min). The SPR response was determined relative to the signal obtained for Milli-Q deionized water.

The main limitation of practical implementation of SPR DNA biosensors is their detection limit, which is too high for the analysis of untreated biological samples. In traditional label-free SPR biosensors that rely on direct DNA hybridization, the typical detection limit is around 50 nM, which is too high for using these devices as a standalone method to detect DNA sequences at physiologically relevant concentrations [23]. Traditional label-free SPR DNA biosensors detect DNA hybridization in real time by monitoring changes in the local refractive index near the metal surface. When single-stranded target DNA binds (hybridizes) to the complementary DNA probe immobilized on the gold surface, it increases the mass density on the surface. This increase in surface mass alters the local refractive index, resulting in a shift of the SPR resonance angle or wavelength, which is directly proportional to the amount of binding. We developed a AuNP-labeled biosensor system in which gold nanoparticles (AuNPs) are used as labels to enhance and amplify the SPR spectrometer signal (Figure 1c). To enhance sensitivity, oligonucleotide probes (SH-DP) complementary to the ssDNA target (80-mer BCR–ABL) are preemptively modified with AuNPs to form the ssDNA-AuNP conjugate (AuNP-SH-DP). Then, the AuNP-SH-DP interacts with the free region of the ssDNA target (80-mer BCR–ABL), which previously bound to immobilized ssDNA probes (mod-Ph). The attachment of AuNP-SH-DP produces a strong signal amplification in SPR due to the substantial molecular mass of AuNPs. As shown in Figure 3, after the addition of AuNP-SH-DP, a more than 10-fold increase in the response of the biosensor to the ssDNA target was observed, due to the hybridization between AuNP-SH-DP and 80-mer BCR–ABL targets.

A key challenge in designing a DNA hybridization assay is to prevent self-hybridization of the probe molecules while maintaining conditions that promote efficient and specific hybridization with the intended target. Therefore, the development of any new DNA detection assay should begin with evaluating the hybridization energies of the relevant oligonucleotide pairs. The principle by which the SPR biosensor allows specific discrimination of DNA targets with the use of a probe oligonucleotide is directly related to the free energy of interaction between these molecules. According to the second law of thermodynamics, among all possible physical processes, those that cause a more significant decrease in the Gibbs free energy (ΔG) in the system are more likely to occur [41].

According to studies on DNA hybridization, more favorable conditions are typically achieved at higher concentrations of positively charged ions, which screen the negative charges of the DNA phosphate backbone, thereby reducing electrostatic repulsion and facilitating strand association and hydrogen-bond formation between complementary bases [42]. This effect is reflected numerically in Table 1, which describes the ΔG values of DNA hybridization in the buffer solutions used in this study.

Table 2 presents the calculated values of the change in the Gibbs free energy of the studied oligonucleotide pairs in SSC buffer solutions of different ionic strengths. All calculations were carried out using the DINAMelt web server with the conditions of 22 °C and a 100 nM concentration of oligonucleotides [43].

Based on the calculated values presented in Table 2, it can be concluded that hybridization of the DNA probes mod-Ph and SH-DP with the 80-mer BCR–ABL target is thermodynamically favored over probe–probe hybridization between mod-Ph and SH-DP. The most negative ΔG values, corresponding to the strongest interactions, were achieved in 2 × SSC buffer. As shown earlier by Matsishin et al., the difference between ΔG values of hybridization of a DNA probe with different targets must be at least two-fold to enable their reliable discrimination by SPR spectrometry [23]. In the case of the 80-mer BCR–ABL target, such a difference ranges from 3.46 to 4.56 relative units.

Another issue that was clarified with the use of the DINAMelt web server was the possibility of detection of BCR–ABL oligonucleotides containing point mutations. In 2 × SSC buffer solutions, the highest value of ΔG of hybridization between mod-Ph and single-mismatched 80-mer BCR–ABL was −30.1, and the highest value of ΔG of hybridization between mod-Ph and single-mismatched 80-mer BCR–ABL was −25.5. This indicates a high affinity of the probes for the target BCR–ABL sequences, even in the presence of point mutations in the target.

The first part of our work was focused on immobilizing SH-DP onto the surface of AuNPs. The average diameter of the modified gold nanoparticles was between 10 and 20 nm. The use of AuNPs of this size is dictated by two factors. First, AuNPs synthesized by the Turkevich method, with an average diameter of ~20 nm or larger, are typically highly polydisperse. This can compromise concentration measurements, because the molar extinction coefficient is calculated using only the mean particle diameter (see Section 2.3) [27]. Secondly, the use of AuNPs with a diameter of less than 4 nm, which have cytotoxic and genotoxic properties, is unacceptable from the point for view of biological safety [44].

**Figure 4:**
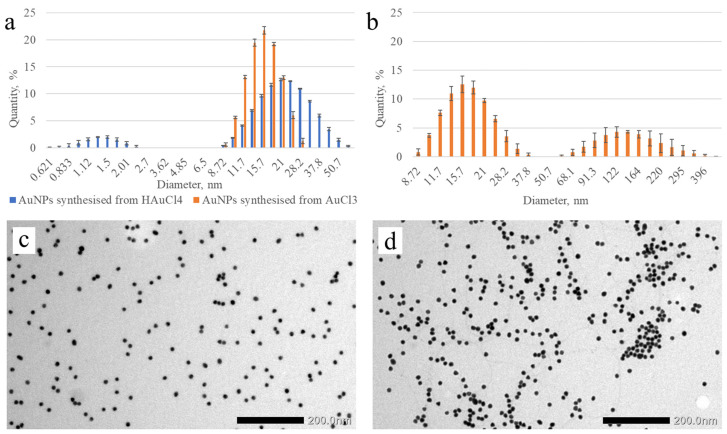
(**a**) Particle diameter distribution of AuNPs synthesized from different gold precursors, plotted as a function of particle diameter. (**b**) Particle diameter distribution of AuNPs synthesized from AuCl_3_ with an additional 10 min of boiling. *x*-axis: AuNP diameter (nm); *y*-axis: fractional light-scattering intensity contributed by AuNPs of a given diameter. (**c**) TEM image of AuNPs synthesized by citrate reduction of AuCl_3_. (**d**) TEM image of AuNPs synthesized by citrate reduction of AuCl_3_ with an additional 10 min of boiling.

In our previous studies, AuCl_3_ was used as source of Au atoms, while HAuCl_4_ is widely used for this purpose in the works of other authors [7,26,27,45]. Therefore, it was decided to investigate the influence of both methods of synthesis of AuNPs on their final size. After the synthesis, the diameter of the obtained AuNPs was estimated by the method of dynamic light scattering using the photon correlation spectroscopy system of the Malvern Zetasizer Nano series. Figure 4 shows the size distributions of AuNPs synthesized by different methods as a function of particle diameter, along with TEM images of AuNPs produced by citrate reduction of AuCl_3_, both without and with an additional 10 min boiling step.

From the size distribution in Figure 4a, it is evident that AuNPs synthesized from HAuCl_4_ differ from those reported by Dong et al. [27]. Specifically, the particles exhibit greater polydispersity, a larger mean diameter, and an additional second peak in the range of diameters up to 4 nm. This heterogeneity, together with the potential toxicity associated with ultrasmall nanoparticles, makes these AuNPs unsuitable for incorporation into the biosensor system. In contrast, substituting HAuCl_4_ with AuCl_3_ yielded monodisperse AuNPs with a size distribution closely matching the literature reports; in particular, the mean particle diameter was 16.39 nm [28]. Accordingly, these AuNPs were selected for incorporation into the biosensor system to enhance its analytical performance.

It was also found that if the reaction mixture was not cooled immediately after the color transition from black to red began, a visible blue precipitate formed in the colloid vessel. The photon correlation spectroscopy results for this AuNP colloid are presented in Figure 4b. It can be seen from the diagram that this sample was distinguished by the presence of a fraction of particles with a diameter of 50–450 nm. This can be explained by the fact that at high temperatures, electrostatic interactions between AuNPs and the carboxylic groups of citric acid are weakened, leading to a decrease in the colloidal stability of such AuNPs and to their coalescence into larger gold aggregates, which have a characteristic blue color. A simple way to prevent this effect is to stop boiling early (i.e., while the colloid is still dark), then incubate the dispersion in the dark at 22 °C for 96 h to allow complete reduction of any remaining Au^3+^ ions.

Next, to enable the reliable use of AuNPs in the biosensor system, their surface was modified to improve colloidal stability and prevent aggregation during their use in the measurement.

The method used in this work promotes the formation of stable covalent Au–S bonds between the AuNP surface and thiol-containing molecules, including mod-Ph and the stabilizing agents 6-mercapto-1-hexanol and lipoic acid. Together, these components provide strong electrosteric repulsion that suppresses AuNP aggregation and thereby enhances colloidal stability [7].

**Figure 5:**
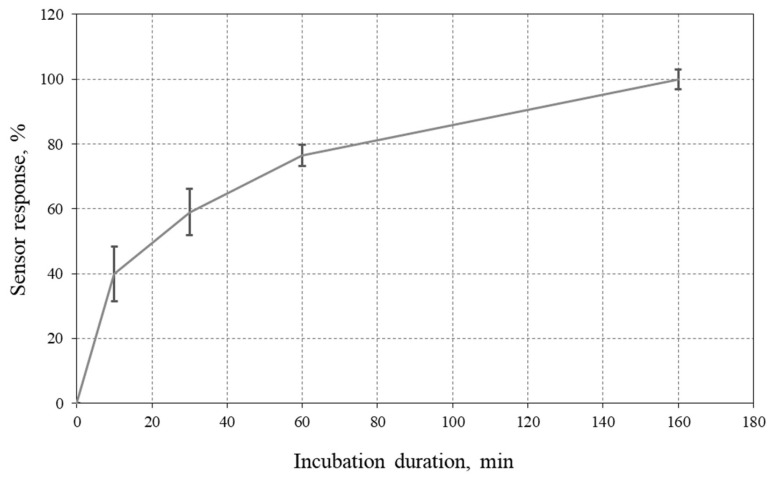
Effect of AuNP–SH–DP incubation time on the SPR biosensor response. Hybridization medium: 2 × SSC buffer solution, concentration of AuNP–SH–DP: 450 pM, concentration of target ssDNA: 100 pM–800 nM. Note: 100% corresponds to the maximum (plateau) signal achieved at the longest incubation time.

The time of AuNP-labeled biosensor response that reached the plateau of the SPR sensogram varied depending on the concentration of the 80-mer BCR–ABL and ranged from 90 to 200 min. Compared to the incubation of the ssDNA target in a traditional label-free SPR biosensor (typically ~10 min), this duration of analysis is exceedingly long. Accordingly, it was necessary to choose a more optimal time of analysis for the developed AuNP-labeled biosensor system. To determine the optimal AuNP–SH–DP incubation time, we compared the reproducibility of AuNP-labeled biosensor signals recorded 10, 30, 60, and 160 min after the start of AuNP–SH–DP incubation with the final plateau response. These AuNP-labeled biosensor responses for different concentrations of 80-mer BCR–ABL (100 pM–800 nM) are shown in Figure 5; 100% corresponds to the maximum biosensor signal that reached the plateau.

According to the obtained data (Figure 5), achieving a standard deviation below 5% requires incubating AuNP–SH–DP for at least 60 min. As shown in Figure 5, the overall analysis time can be substantially reduced, at the expense of accuracy. Therefore, all subsequent hybridization experiments were carried out using a fixed incubation time of 60 min.

In the present work, the 60 min incubation time was selected to ensure sufficiently complete hybridization and to maximize signal amplification, which was the primary objective of this proof-of-concept study. However, we acknowledge that further optimization of this step is essential for practical applications. Potential strategies for reducing the incubation time include: (i) increasing the effective concentration of the AuNP-conjugated probes; (ii) optimizing the buffer composition and ionic strength to accelerate hybridization kinetics; (iii) employing temperature-assisted hybridization; and (iv) introducing active mixing or microfluidic flow to enhance mass transport. In addition, the AuNP-enhanced format provides a substantial improvement in detection sensitivity compared with the label-free assay, which may justify the longer analysis time, depending on the application requirements.

**Figure 6:**
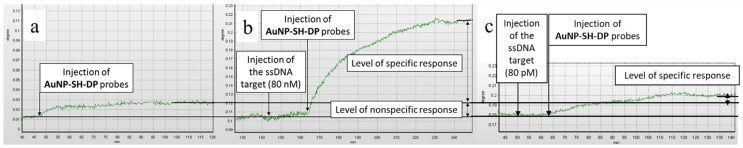
(**a**) Non-specific biosensor response in the absence of ssDNA targets in the measuring cell; (**b**) biosensor response to injection of the 80-mer BCR–ABL DNA target (80 nM) into the measuring cell, followed by injection of AuNP–SH–DP; (**c**) biosensor response to the introduction of the 80-mer BCR–ABL DNA target (80 pM) into the measuring cell, followed by injection of AuNP–SH–DP. Hybridization medium: 2 × SSC; concentration of AuNP-SH-DP: 450 pM; *y*-axis: SPR response (degrees, °); *x*-axis: time (min). SPR response is measured relative to the response of Milli-Q deionized water.

In order to analyze the specificity of the proposed AuNP–labeled biosensor system, experiments were conducted with the absence and presence of the 80-mer BCR–ABL DNA target in the studied samples. The sensograms of such measurements are shown in Figure 6.

Using Figure 6b as an example, the biosensor’s specific response to a given concentration of the 80-mer BCR–ABL DNA target (80 nM) was calculated by subtracting the non-specific response (0.017°) from the measured signal (0.107°). Accordingly, the specific response of the AuNP-labeled biosensor to the 80-mer BCR–ABL DNA target (80 nM) in this case was 0.090°.

The AuNP-labeled SPR signal enhancement can be obtained only when both hybridization events occur (i) between the mod-Ph probe and the target (BCR sequence) and (ii) between the Au-SH-DP probe and the target (ABL sequence). Therefore, if the injected target contains the BCR sequence but not the ABL sequence (or vice versa), the sandwich hybridization cannot take place, and the AuNPs will not approach the SPR sensor surface and will not enhance the signal.

This principle also supports the expected selectivity of the developed AuNP-enhanced SPR biosensor toward the BCR–ABL hybrid sequence. In particular, oligonucleotides corresponding to the normal BCR or ABL genes alone would not be expected to generate an enhanced SPR response, because only one of the two required hybridization events could take place. As a result, the full sandwich complex cannot be formed, the AuNPs remain distant from the sensor surface, and the response is expected to be similar to that observed in the absence of target DNA.

Future studies will include a more detailed evaluation of selectivity using non-complementary and mismatched sequences, together with validation in complex biological matrices. In the present work, the primary objective was to demonstrate the feasibility of the proposed AuNP-enhanced SPR biosensor and the effectiveness of its signal amplification strategy. Accordingly, the experimental validation was restricted to model conditions.

**Figure 7:**
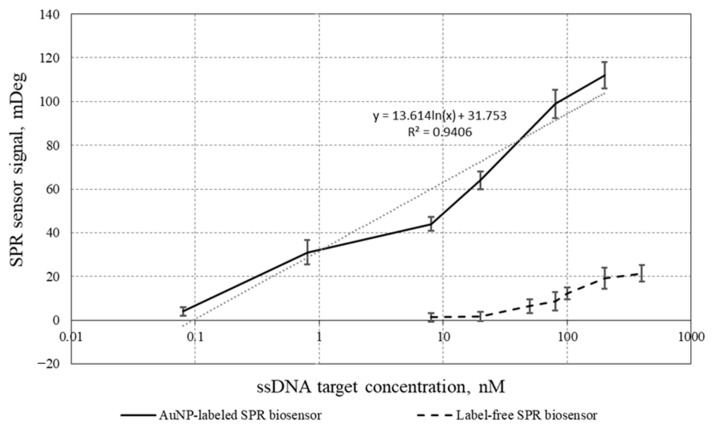
Calibration curves for quantifying the 80-mer BCR–ABL target in solution using the AuNP-labeled SPR biosensor and the label-free SPR biosensor. Hybridization medium: 2 × SSC buffer solution; concentration of AuNP-SH-DP: 450 pM; *y*-axis: specific SPR response (mDeg); *x*-axis: concentration of the 80-mer BCR–ABL target (nM). The SPR response was determined relative to the signal obtained for Milli-Q deionized water.

Figure 7 presents the calibration curves of two biosensors: the AuNP-labeled SPR biosensor (sandwich type) and the label-free SPR biosensor (traditional). According to the obtained results, the use of AuNPs as labels markedly increases the SPR biosensor response and significantly decreases the LOD for ssDNA target detection.

As shown in Figure 7, the AuNP-labeled SPR biosensor exhibits a linear response on a logarithmic scale over the 80-mer BCR–ABL target concentration range of 80 pM to 200 nM.

**Figure 8:**
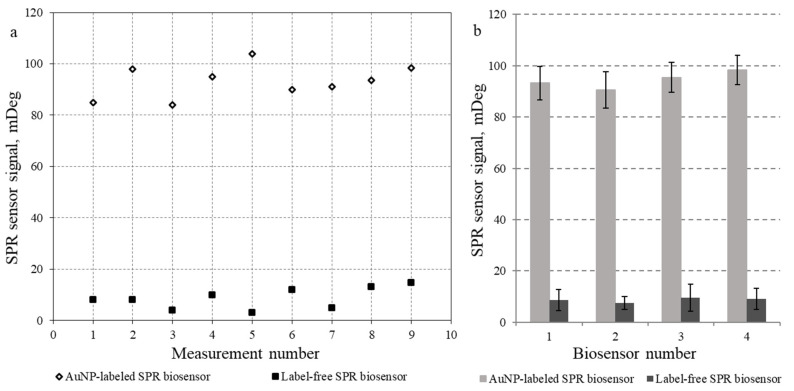
Reproducibility of SPR biosensor signals for detection of 80-mer BCR–ABL DNA target at a concentration of 80 nM. (**a**) Nine distinct biosensor responses obtained with AuNPs from two different batches (measurements 1–4 and 5–9). (**b**) Mean values and standard deviations of responses of three different SPR biosensors. Hybridization medium: 2 × SSC, concentration of AuNP-SH-DP: 450 pM.

To evaluate the signal reproducibility of the proposed AuNP-labeled biosensor for detecting the BCR–ABL hybrid gene; we recorded nine SPR responses after introducing the same concentration (80 nM) of the 80-mer BCR–ABL target into the measurement flow cell (Figure 8). The results demonstrate high reproducibility of the developed AuNP-labeled SPR biosensor system.

To assess potential batch-to-batch signal variation, the AuNPs used for response enhancement were taken from two independently prepared batches (Figure 8a). No appreciable differences in reproducibility were observed between batches (batch 1: 90.5 ± 7.1; batch 2: 95.4 ± 5.8; mean ± SD).

As shown in Figure 8a, the AuNP-labeled SPR biosensor produced highly reproducible responses, with a relative standard deviation (RSD) of 6.96%, whereas the label-free SPR biosensor detected the same target DNA concentration with a substantially higher variability (RSD = 43.64%). The overall RSD of the developed AuNP-labeled SPR biosensor is equal to 6.64% (Figure 8b). Although the measurement error of the label-free format decreases at higher target concentrations, SPR biosensors are typically applied in scenarios where achieving the lowest possible detection limit is critical. The reproducibility may be further improved using SPR instruments equipped with a thermally controlled flow cell and an automated measurement procedure. In agreement with earlier findings, the complete number of biosensor regeneration cycles before performance degradation was found to be no lower than 50 [7].

Compared with the label-free SPR biosensor [23], the proposed AuNP-labeled SPR biosensor for detecting the BCR–ABL sequence (Philadelphia chromosome) enabled quantification of the 80-mer BCR–ABL target with substantially higher sensitivity and a markedly lower detection limit. The limit of detection (LOD) for the BCR–ABL target using the AuNP-labeled SPR biosensor was 80 pM, which is 625-fold lower than the LOD of the label-free SPR biosensor (50 nM). At a target DNA concentration of 80 nM, the response of the AuNP-labeled SPR biosensor was more than tenfold higher than that of the label-free SPR biosensor. This makes the developed AuNP-labeled SPR biosensor more promising for determining the BCR–ABL DNA target in real biological samples. In addition, when working at low target concentrations, the AuNP-labeled SPR biosensor exhibited a substantially lower measurement error comparing with the label-free SPR biosensor.

## 4. Conclusions

We developed a novel approach for e13a2 BCR–ABL gene sequence detection by designing an AuNP-labeled, DNA hybridization–based, sandwich-type SPR biosensor. We showed that using probe oligonucleotides conjugated to stabilized AuNPs in an SPR-based biosensor assay can increase the specific sensor response to BCR–ABL gene oligonucleotides by several fold.

A novel citrate-reduction method for synthesizing AuNPs from AuCl_3_ was developed to produce highly monodisperse gold nanoparticles with an optimal diameter for biosafe applications.

The effect of AuNP–SH–DP incubation time on the SPR biosensor response was investigated. Reproducible sensor responses were achieved at incubation times of 60 min. Although this increases the total assay time, the AuNP-enhanced format provides substantially improved detection sensitivity compared with the label-free SPR assay, which may justify the longer analysis time depending on the application. Further reduction of the incubation time may be achieved by optimizing probe concentration, hybridization conditions, and mass transport.

It was established that the developed AuNP-labeled SPR biosensor achieved a detection limit of 80 pM, which is more than 500-fold lower than that of the traditional label-free SPR biosensor. The AuNP-labeled SPR biosensor provides a linear detection range on a logarithmic concentration scale for the BCR–ABL target of 80 pM to 200 nM, making it a promising approach for determining BCR–ABL DNA in real biological samples. The developed AuNP-labeled DNA biosensing approach can be adapted to enhance the signal in other DNA hybridization-based SPR biosensors, particularly in assays for simultaneous detection of multiple variants of the BCR–ABL gene (e.g., e13a2 and e14a2).

## Figures and Tables

**Table 1:** A comparison of qPCR assays and SPR biosensors for the detection of BCR–ABL sequences [18,21,22,23].

Characteristics	qPCR	SPR
Analysis time	~2–2.5 h	~1–2 h
LOD	~0.003% IS (few copies per reaction)	~10 nM (classical label-free SPR)

**Table 2:** Calculated Gibbs free energy changes (ΔG, kcal/mol) for hybridization of the studied oligonucleotide pairs in SSC buffer solutions of different ionic strengths.

Oligonucleotide Pair	ΔG in 0.1 × SSC (kcal/mol)	ΔG in 0.2 × SSC (kcal/mol)	ΔG in 2 × SSC (kcal/mol)
mod-Ph and SH-DP	−5.4	−6.0	−8.0
mod-Ph and 80-mer BCR–ABL	−28.8	−30.6	−36.5
80-mer BCR–ABL and SH-DP	−22.1	−23.4	−27.7

## Data Availability

The original data presented in the study are openly available in Open Science Framework at https://osf.io/y6vfs/overview?view_only=728f9b28ac284e188004be64b0a7ea85 (accessed on 18 February 2026).
